# Antidiabetic, anticholinergic and antioxidant activities of aerial parts of shaggy bindweed (*Convulvulus betonicifolia* Miller subsp.) – profiling of phenolic compounds by LC-HRMS

**DOI:** 10.1016/j.heliyon.2021.e06986

**Published:** 2021-05-08

**Authors:** Zeynebe Bingol, Hatice Kızıltaş, Ahmet C. Gören, Leyla Polat Kose, Meryem Topal, Lokman Durmaz, Saleh H. Alwasel, İlhami Gulcin

**Affiliations:** a Department of Chemistry, Faculty of Sciences, Atatürk University, Erzurum 25240, Turkey; b Vocational School of Health Services, Van Yuzuncu Yil University, Van 65080, Turkey; c Department of Analytical Chemistry, Faculty of Pharmacy, Bezmialem Vakif University, Istanbul 34093, Turkey; d Drug Application and Research Center, Bezmialem Vakif University, Istanbul 34093, Turkey; e Vocational School, Department of Pharmacy Services, Beykent University, Buyukcekmece, Istanbul 34500, Turkey; f Vocational School of Health Services, Gumushane University, Gumushane 29000, Turkey; g Department of Medical Services and Technology, Cayirli Vocational School, Erzincan Binali Yildirim University, Cayirli, Erzincan 24500, Turkey; h Department of Zoology, College of Science, King Saud University, Riyadh, Saudi Arabia

**Keywords:** Shaggy bindweed, *Convulvulus betonicifolia*, Antioxidant activity, Polyphenol content, LC-HRMS

## Abstract

In order to evaluate the antioxidant activity of evaporated ethanolic extract (EESB) and lyophilized water extract (WESB) of Shaggy bindweed (*Convulvulus betonicifolia* Mill. Subs), some putative antioxidant methods such as DPPH· scavenging activity, ABTS^•+^ scavenging effect, ferric ions (Fe^3+^) reduction method, cupric ions (Cu^2+^) reducing capacity, and ferrous ions (Fe^2+^) binding activities were separately performed. Also, ascorbic acid, α-tocopherol and BHT were used as the standard compounds. Additionally, some phenolic compounds that responsible for antioxidant abilities of EESB and WESB were screened by liquid chromatography-high resolution mass spectrometry (LC-HRMS). At the same concentration, EESB and WESB demonstrated effective antioxidant abilities when compared to standards. In addition, EESB demonstrated IC_50_ values of 1.946 μg/mL against acetylcholinesterase (AChE), 0.815 μg/mL against α-glycosidase and 0.675 μg/mL against α-amylase enzymes.

## 1 Introduction

Shaggy bindweed (*Convulvulus betonicifolia* Mill. Subs) is endemic plant to Eastern Anatolia. *Convolvulus* species are used as a laxative in Anatolian folk medicine. Also, in some regions of Anatolia, convolvulus leaves are added to soup in kitchens [1]. This plant is a hairy herbaceous perennial that is drifting or climbing. Its leaves and flowers are solitary, axillary or in 2-5-flowered cymes. Its outer sepals hairy, oblong, sharp to acuminous. It usually grows in fallow or cultivated fields, along roadsides, in dry ditches and at 30–1700 m altitude.

The plants are known as productive and prolific haven of phytochemicals with unmatched therapeutic potentials. Moreover, worldwide 28.000 plant taxa have been reported to have medicinal properties. It has been recorded that more than 3000 species have ethnomedical applications against many diseases including cancer [2]. According to recent studies, the use of endemic and medicinal plants in the food, pharmaceutical and cosmetic industries is recently increasing. Meanwhile, the majority of the world's population frequently uses herbal medicine for basic health care [3]. Medicinal plants are an important source of nutrients and organic metabolites to protect human health. They are commonly used in developing countries and around the world, to treat some diseases especially in metabolic syndrome and diabetes mellitus [4]. It was reported that medicinal plants have many crucial pharmacological and biological effects such as antioxidant, anti-inflammatory, anticancer, and others. It is well known that these plants have antioxidant effects and serve as sources of phenolic compounds [5, 6].

Antioxidant molecules are natural or synthetic substances, which inhibit oxidation process and blocked the production of free radicals and reactive oxygen species (ROS) [7, 8]. They can preserve the human body from undesired effects of ROS and oxidative stress [9, 10]. Antioxidants had beneficial effects in preventing of chronic diseases. They can easily terminate the radical chain reactions and neutralize free radicals, which attack biomolecules in cells and tissues [11, 12]. Also, antioxidants are used as additives to protect food products against rancidity, color and texture loss, and extend shelf life by preventing unwanted odors. Also, it was demonstrated that some synthetic antioxidants, which used in the foods are toxic and had carcinogenic effects [13]. Otherwise, vegetables and fruits have a wide range of antioxidants and are a rich source of healthy food. In this regard, most antioxidant molecules from natural sources such as plants have been found as ROS or free radical scavengers [14, 15]. For this reason, alternative, natural and reliable plant-derived antioxidants are preferred as natural antioxidants [16, 17]. The overall antioxidant potential of a specific compound or herbal extract, whose reactivity to different ROS can vary continuously, depends on many parameters such as free radical scavenging capacity, reducing strength and redox buffering activity, lipid peroxidation inhibitor and metal chelating properties [18].

Antioxidants delay or avoid the onset of major degenerative diseases such as diabetes mellitus and AD [17, 19, 20]. One of the main targets in the treatment diabetes is the α-glycosidase that its activity is fundamental to the degradation of dietary polysaccharides. α-Glucosidase inhibitors (AGIs) prevent the breakdown of polysaccharides into monosaccharide units and thus block the absorption of monomeric sugars in the intestinal tract. In this way, it limits the postprandial plasma glucose level. AGIs can be used to treat diabetes mellitus (DM) and obesity [21, 22].

AD is the most typical and widespread form of dementia among older people that negatively affects the ability to perform personal daily activities. It is also well known that cholinergic conduction loss is one of the main causes of AD [23]. Therefore, acetylcholinesterase inhibitors (AChEIs) that enhance cholinergic transmission can be used to treat AD. Among them, tacrine is currently used in the palliative therapy for mild to moderate AD patient as AChEI. It is known that most of these drugs have undesired and common side effects such as nausea, headache, vomiting and diarrhea [24, 25]. Also, these clinical used inhibitors exhibit undesirable side effects such as hepatotoxicity and gastrointestinal anomalies including nausea and diarrhea [26, 27]. Therefore, there is a great demand to develop and use AChEIs that are new and known for their antioxidant properties. With all this, it is well established that polyphenols also have anti-AD properties and α-glycosidase inhibition profiles. Therefore, one of the most important therapy approaches for neurodegenerative diseases and DM is natural compounds and products including phenolic compounds [28, 29, 30, 31]. However, current evidence demonstrates that patients with DM have an increased risk of developing AD. These evidences also show that hyperinsulinemia and insulin resistance-T2DM are distinguishing features [32].

In this study, the Cu^2+^, Fe^3+^ and FRAP reducing abilities, DPPH˙ and ABTS˙^+^ scavenging and Fe^2+^ chelating activities of EESB and WESB were investigated. A noteworthy feature of the study is to quantitatively elucidate some important phenolic contents in plant extracts with LC-HRMS chromatography. Also, another main goal of this study was to determine the possible inhibition effects of EESB and WESB against acetylcholinesterase and α-glycosidase linked to Alzheimer's disease and diabetes.

## 2 Materials and methods

### 2.1 Chemicals

α-Tocopherol, neocuproine, DPPH radical, ABTS and DMPD, Trolox and α-Tocopherol were obtained from Sigma-Aldrich (Germany). The other chemicals were used for analytical grade and purchased from Merck or Sigma-Aldrich.

### 2.2 Plant materials

Shaggy bindweed (*Convolvulus betonicifolius* Mill. subsp. *peduncularis* (Boiss.) Parris Summerh) was collected from 2 km South of Çaldıran, B9 Van: Çaldıran, 2200 m, in June 2019 (location: 39°06′46.0″N 43°51′12.8″E, MP 16438 code). The plant was identified by botanist Dr. Süleyman Mesut Pınar, Van Yüzüncü Yıl University. Plant samples were deposited at Van Yüzüncü Yıl University, Faculty of Science, Herbarium of the Biology Department (VANF), Van, Turkey.

### 2.3 Extracts preparation

Water and ethanol extractions methods were described previously [33]. For determination of the ethanolic extract of aerial parts of shaggy bindweed (*Convulvulus betonicifolia* Mill. Subs), a 50 g plant sample was cut into small pieces, then, grind it into powder, mixed with 0.5 L of ethyl alcohol and evaporated [34]. This process was repeated until the color of extraction solution turned to colorless. The combined extracts were filtered (Whatman paper) and evaporated (Heidolph Hei-VAP HL, Germany). Dried ethanolic extract of shaggy bindweed (*Convulvulus betonicifolia* Mill. Subs) (EESB) was transferred to an appropriate glass bottle and stored (–20 °C) until used in experiments.

For lyophilized water extraction, a 50 g shade-dried shaggy bindweed (*Convulvulus betonicifolia* Mill. Subs) samples powdered and mixed with 0.5 L deionized water, boiled and stirred for 20 min. Then, extract was filtered and frozen in an ultra-low temperature freezer (-87 °C). The frozen extract was lyophilized (-50 °C and 5 mm-Hg) in a lyophilizator [35]. Next, the prepared fresh lyophilized water extract of shaggy bindweed (*Convulvulus betonicifolia* Mill. Subs) (WESB) was stored in a plastic bottle and stored at –20 °C until used in experimental.

### 2.4 Reducing ability assays

The ferric ions (Fe^3+^) reducing ability of EESB and WESB were performed according to method of Oyaizu [36] as given previously [37, 38]. Briefly, different amounts of EESB and WESB in water or ethanol (10–50 μg/mL) were added to the equal volume of phosphate buffers (pH 6.6, 1.25 mL, 0.2 mol/L) and K_3_Fe(CN)_6_ solution (1%, 1.25 mL). The solution was kept at 50 °C during 20 min and then, acidified with TCA (10%, 1.25 mL). Finally, a portion of FeCl_3_ (0.1%, 0.5 mL) was added and their absorbances were spectrophotometrically recorded at 700 nm.

The cupric ions (Cu^2+^) reducing effects of EESB and WESB were determined according to spectrophotometric assay [39] as described in details [40]. For this aim, the equal volumes of CuCl_2_ (250 μL, 10 mmol/L), neocuproine (7.5 mmol/L) and acetate buffer (0.25 mL, 1.0 mol/L) were used. Finally, their absorbances were spectrophotometrically recorded at 450 nm [41].

FRAP reduction ability was realized according to our previous study [42]. The former stock solution was used for this experiment was used for this assay. A portion (2.25 mL) of TPTZ solution (10 mmol/L TPTZ in 40 mmol/L HCl) was freshly prepared, then transferred to 2.5 mL acetate buffer (0.3 mol/L, pH 3.6) and 2.25 mL of FeCl_3_ solution (20 mmol/L) were used. Finally, the absorbance of samples was spectrophotometrically measured at 593 nm.

### 2.5 Radical scavenging activities

DPPH∙ scavenging ability of EESB and WESB was performed according to Blois method [43] as given in prior study [44, 45]. The N-centered DPPH radicals was used for the estimation of the radical scavenging capacity of plant extracts. In brief, an aliquot of DPPH radicals (0.5 mL, 0.1 mmol/L) was added to EESB and WESB solutions. The absorbance of the mixtures was recorded at 517 nm.

ABTS^∙+^ scavenging ability of EESB and WESB was performed according to the previous study [46]. Primarily an ABTS solution (7.0 mmol/L) was produced by adding to K_2_S_2_O_8_ and their absorbances was set to 0.750 ± 0.025 nm diluted. The absorbance of samples was spectrophotometrically recorded at 734 nm.

The radical scavenging abilities (RSA) of the EESB and WESB were found as millimolar in the reaction mixture. Both radicals (DPPH^•^ and ABTS^•+^) scavenging effects were calculated from Eq. (1).

(1) RSA (%) = (1-A_A_/A_B_) × 100

where A_A_ and A_B_ are the absorbances of the control and samples, respectively [47].

### 2.6 Anticholinergic assay

The AChE inhibitory effects of EESB and WESB was realized according to Ellman's method [48] as given in previous studies [49, 50]. AChE was obtained from electric eel and DTNB and acetylthiocholine iodide (AChI) were used as substrates for cholinergic reaction [51]. Namely, 0.1 mL of Tris/HCl buffer (pH 8.0, 1.0 mol/L), different concentrations of EESB and WESB were prepared in ethanol and deionized water. Then, 50 μL of AChE (5.32 × 10^−3^ EU) solution was transferred and incubated (20 min at 25 °C). After a short incubation period, 50 μL of DTNB (0.5 mmol/L) was added and the reaction was started by the addition of 50 μL of AChI (10 mmol/L). The absorbances were recorded at 412 nm.

### 2.7 Antidiabetic assay

Both digestive enzymes inhibition effects of EESB and WESB were studied within the scope of the antidiabetic study. α-Glycosidase inhibition efficacy of EESB and WESB was performed according to Tao's method [52]. p-Nitrophenyl-D-glucopyranoside (p-NPG) was used as substrate. The absorbances were spectrophotometrically measured at 405 nm [49]. First, 75 μL of phosphate buffer was added with 20 μL of the α-glycosidase solution (0.15 U/mL) and 50 μL of different concentrations EESB and WESB, which dissolved in ethanol and deionized water, respectively. Then, it was preincubated at 35 °C for 20 min prior to the adding of p-NPG to the initiation of the reaction. In addition, 20 μL of p-NPG was added in phosphate buffer (5 mmol/L and pH 7.4) after preincubation at 35 °C. The absorbance was measured at 405 nm [53].

α-Amylase enzyme activity was determined according to the Xiao's procedure [54]. Starch was used as substrate and prepared in NaOH solution (80 mL, 0.4 mol/L, 30 min and 80 °C). The 50 μL of starch solution, 50 μL of phosphate buffer (pH 6.9), and 50 μL of different concentration EESB and WESB sample dissolved in ethanol and deionized water were mixed and was preincubated at 35 °C for 30 min. Then, 30 μL of α-amylase solution was transferred and kept for 30 min. The absorbances were recorded at 580 nm.

### 2.8 Determination of inhibition parameters

The IC_50_ was obtained from enzyme activity (%) versus and plant concentration plots [55]. Determination of IC_50_ values were described previously [56].

### 2.9 Total phenolic and flavonoid contents

Total phenolics in EESB and WESB were calculated by Folin-Ciocalteu methods [57] as descried in prior studies [58] and calculated from gallic acids calibration curve. The results are calculated as μg gallic acids equivalents (GAE) per g plant extract. In addition, total flavonoids in EESB and WESB were calculated according our previous colorimetric method [59] and calculated from standard quercetin curve. The results are given as μg quercetin equivalents (QE) per g plant extract.

### 2.10 Preparation of samples for LC-HRMS analysis

The dried 50–100 mg of the EESB and WESB were dissolved in water in a 5 mL volumetric flask, which was stored in an ultrasonic bath until obtained a clear solution. Then, 0.1 mL of dihydrocapsaicin solution that used as an internal standard was diluted with mobile phase and heated, stirred and filtered. The final solution (1 mL) was added in a capped auto sampler vial, from which 2 μL of sample was injected to LC for each run. The prepared samples in the auto sampler were stored at 15 °C [60, 61, 62, 63].

### 2.11 Instruments and chromatographic conditions of LC-HRMS

LC-HRMS experiments were achieved on a Thermo ORBITRAP Q-EXACTIVE mass spectrometry equipped with a Troyasil C18 column (150 × 3 mm i.d., 3 μm particle size). The mobile phases A and B were composed of formic acid-water (1%) and formic acid-methanol (1%), respectively. The gradient program of which was 0–1.00 min 50% A and 50% B, 1.01–6.00 min 100% B, and finally 6.01–10 min 50% A and 50% B. The flow rate of the mobile phase was 0.35 mL/min, and the column temperature was set to 22 °C. Environmental conditions were set as temperature 22.0 ± 5.0 °C and relative humidity (50 ± 15) % rh [62].

### 2.12 Optimization of HPLC and LC-HRMS procedures

The best mobile phase was performed to be an acidified with methyl alcohol and water gradient in HPLC method. This mobile phase was also found as suitable for ionization abundance and separation of compounds. The best ionization of relatively polar and small compounds was found by ESI source. The ions between m/z 85–1500 were scanned in high-resolution mode of instrument [60, 61, 62, 63]. The MS parameters are used as follows: sheath gas flow rate: 45, aux gas flow rate 10, spray voltage (kV): 3.80, capillary temperature (°C): 320, aux gas heater temp (°C): 320 and S-lens RF level:50. Identification of compounds was performed by comparison of retention time of standards (in the range of purity 95–99% see section chemicals) and HRMS data of Bezmialem Vakif University, Drug Application and Research Center Library (ILMER). Dihydrocapsaicin (purity 95%) was used as an internal standard for LC-HRMS measurements in order to reduce to repeatability problem of caused by external effects, such as ionization repeatability, in mass spectrometry measurements. The detailed mass parameters of each target compounds were given in Table 1.

**Table 1:** LC-HRMS parameters of selected compounds.

Compounds	m/z	Ionization mode	Linear range (mg/mL)	Linear regression equation	LOD/LOQ (mg/mL)	R^2^	Recovery
Ascorbic acid	175.0248	Negative	0.5–10	y = 0.00347x−0.00137	0.39/1.29	0.9988	96.20
(−)-Epigallocatechin	307.0812	Positive	0.3–5	y = 0.00317x + 0.000443	0.17/0.57	0.9947	102.22
Chlorogenic acid	353.0878	Negative	0.05–10	y = 0.00817x + 0.000163	0.02/0.06	0.9994	96.68
Verbascoside	623.1981	Negative	0.1–10	y = 0.00758x + 0.000563	0.03/0.1	0.9995	96.19
Orientin	447.0933	Negative	0.1–10	y = 0.00757x + 0.000347	0.01/0.03	0.9993	96.22
Caffeic acid	179.0350	Negative	0.3–10	y = 0.0304x + 0.00366	0.08/0.27	0.9993	94.51
Luteolin-7-rutinoside	593.1512	Negative	0.1–10	y = 0.00879x + 0.000739	0.01/0.03	0.9988	93.05
Naringin	579.1719	Negative	0.05–10	y = 0.00576x−0.000284	0.01/0.03	0.9991	101.91
Luteolin 7-glucoside	447.0933	Negative	0.1–7	y = 0.0162x + 0.00226	0.01/0.03	0.9961	96.31
Hesperidin	609.1825	Negative	0.05–10	y = 0.00423x + 0.0000138	0.01/0.03	0.9994	96.14
Rutin	609.1461	Negative	0.05–10	y = 0.00329x−0.00005576	0.01/0.03	0.999	96.97
Syringic acid	197.0456	Negative	0.5–10	y = 0.0000831x + 0.000024	0.1/0.3	0.9991	97.29
Rosmarinic acid	359.0772	Negative	0.05–10	y = 0.00717x−0.0003067	0.01/0.03	0.9992	99.85
Hyperoside	463.0882	Negative	0.05–10	y = 0.0072x−0.00003096	0.01/0.03	0.9995	96.62
Apigenin 7-glucoside	431.0984	Negative	0.3–7	y = 0.0246x + 0.00306	0.01/0.03	0.9962	96.07
Quercitrin	447.0933	Negative	0.05–10	y = 0.0179 + 0.0003331	0.01/0.03	0.999	97.00
Quercetin	301.0354	Negative	0.1–10	y = 0.0509x + 0.00467	0.01/0.03	0.9978	96.41
Salicylic acid	137.0244	Negative	0.3–10	y = 0.0361x + 0.00245	0.01/0.03	0.9982	92.88
Naringenin	271.0612	Negative	0.1–10	y = 0.0281x + 0.00182	0.01/0.03	0.9995	86.65
Luteolin	285.0405	Negative	0.1–10	y = 0.117x + 0.00848	0.01/0.03	0.9981	96.98
Apigenin	269.0456	Negative	0.3–10	y = 0.104x + 0.0199	0.01/0.03	0.9998	81.55
Hispidulin	301.0707	Positive	0.05–10	y = 0.02614x + 0.0003114	0.01/0.03	0.9993	98.36
Isosakuranetin	285.0769	Negative	0.05–10	y = 0.0235x + 0.000561	0.01/0.03	0.9992	96.56
Chrysin	253.0506	Negative	0.05–7	y = 0.0964x−0.0002622	0.01/0.03	0.999	87.92
Acacetin	283.0612	Negative	0.05–7	y = 0.046x + 0.0001875	0.01/0.03	0.9995	87.52

“<LOD”: These values are below the limits of the quantification.

## 3 Results and discussion

**Figure 1:**
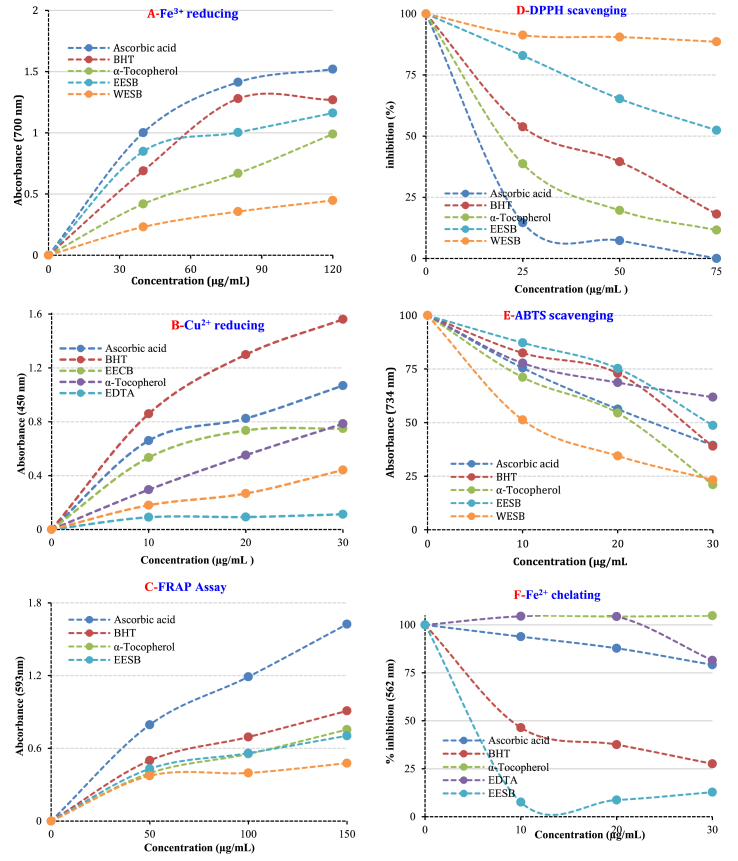
Antioxidant activities of EESB, WESB and reference antioxidants. A. Fe^3+^ reducing power. B. Cu^2+^ reducing power, C. Fe^3+^-TPTZ reducing power, D. DPPH radical scavenging, E. ABTS radical scavenging, F. Metal chelating activity [EESB: Evaporated ethanolic extract of aerial parts shaggy bindweed (*Convulvulus betonicifolia* Mill. Subs). WESB: Lyophilized water extract of aerial parts of shaggy bindweed (*Convulvulus betonicifolia* Mill. Subs), BHT: butylated hydroxytoluene].

Natural compounds from different plant sources have been vital importance in food and other industries. Most of modern medicines are based on natural derivatives. It has been observed that the consumption of antioxidants from natural products such as nutritious herbs, herbs and spices are beneficial for human health and had effective in reducing the ROS occurrence and oxidative stress-related diseases [64]. Antioxidant properties of EESB and WESB have been carried out in several bioanalytical methods such as Fe^2+^ chelating activity, Fe^3+^ reducing activity, Fe^3+^-TPTZ reduction capacity, Cu^2+^ reduction ability, ABTS and DPPH radicals scavenging activities [18]. For comparison of antioxidant effects, putative standard compounds of α-tocopherol, BHT and ascorbic acid were used. It was found that the antioxidant activities of EESB and WESB are similar or close to standards. It was shown that the antioxidant ability of EESB and WESB enhanced with increasing concentration (10–30 μg/mL). Moreover, the antioxidant ability of EESB and WESB was observed to be higher than some standard antioxidants at the same concentration, in some cases. Reducing ability is one of the most significant factors in its total antioxidant effectiveness [[65], [66]]. The antioxidant activity of a molecule or plant extract can occur using different mechanisms [67]. Antioxidants may be in the form of stabilizing oxidants in reductant and redox reactions. The reduction capacity can be recorded by diverse bioanalytical methods. In the presence of reducing compounds, the reduction of ferric complex (Fe[(CN)_6_]^3+^) to the ferrous form (Fe[(CN)_6_]^2+^) can easily occur. The addition of Fe^3+^ to the reduced product by addition of EESB and WESB leads to Fe_4_[Fe(CN)_6_], a complex in the Prussian blue color with sharp absorbance at 700 nm [68]. The increased absorbance shows the increased reduction capacity. As seen in Table 2, EESB and WESB exhibited potent Fe^3+^ reducing effects. These diversities were statistically found to be considerable important (p < 0.01). The reducing capacity of EESB and WESB, α-tocopherol, BHT, and ascorbic acid increased constantly when the concentration of sample was increased in following order: Ascorbic acid (λ_700_: 1.520 ± 0.028, r^2^: 0.9970) > BHT (λ_700_: 1.269 ± 0.005, r^2^: 0.9880) > EESB (λ_700_: 1.161 ± 0.008, r^2^: 0.9683) > α-Tocopherol (λ_700_: 0.990 ± 0.007, r^2^: 0.9942) > WESB (λ_700_: 0.447 ± 0.011, r^2^: 0.9977) at 30 μg/mL. The results exhibited that EESB and WESB had noteworthy Fe^3+^ reducing effect (Figure 1A and Table 2).

**Table 2:** The reducing power of the EESB and WESB and standards antioxidants by Fe^3+^-Fe^2+^ (30 μg/mL), Cu^2+^-Cu^+^ (30 μg/mL) and Fe^3+^-TPTZ (50 μg/mL) reducing methods (EESB: Evaporated ethanolic extract of aerial parts shaggy bindweed (*Convulvulus betonicifolia* Mill. Subs). WESB: Lyophilized water extract of aerial parts of shaggy bindweed (*Convulvulus betonicifolia* Mill. Subs).

Antioxidants	Fe^3+^-Fe^2+^ reducing		Cu^2+^-Cu^+^ reducing		Fe^3+^-TPTZ reducing	
	λ_700_	r^2^	λ _450_	r^2^	λ _593_	r^2^
α-Tocopherol	0.990 ± 0.007	0.9942	0.785 ± 0.061	0.9986	0.755 ± 0.075	0.9867
Ascorbic acid	1.520 ± 0.028	0.9970	1.069 ± 0.007	0.9722	1.624 ± 0.015	0.9930
BHT	1.269 ± 0.005	0.9880	1.561 ± 0.089	0.9978	0.909 ± 0.006	0.9874
EESB	1.161 ± 0.008	0.9683	0.749 ± 0.036	0.9970	0.704 ± 0.005	0.9808
WESB	0.447 ± 0.011	0.9977	0.441 ± 0.037	0.9833	0.477 ± 0.018	0.9359

Another putative and common method is Fe^3+^-TPTZ reduction assay, which based on the reducing of the Fe^3+^-TPTZ complex to Fe^2+^-TPTZ [69]. As seen in Figure 1C and Table 2, the FRAP reducing ability of EESB and WESB declined as follows: Ascorbic acid (λ_593_: 1.624 ± 0.015, r^2^: 0.9930) > BHT (λ_593_: 0.909 ± 0.006, r^2^: 0.9874) > α-Tocopherol (λ_593_: 0.755 ± 0.075, r^2^: 0.9867) > EESB (λ_593_: 0.704 ± 0.005, r^2^: 0.9808) and WESB (λ_593_: 0.477 ± 0.018, r^2^: 0.9359) at 50 μg/mL. The FRAP assay is carried out in an acidic environment for iron solubility [38].

Copper is an important element in metallic form, which can be found and used directly in nature. It is a very important cofactor for some endogenous and metabolic enzymes such as cytochrome c oxidase [70]. Also, this chromogenic redox reaction is used to determine the potential of antioxidants containing non-protein thiols and thiols such as glutathione. Cupric ions (Cu^2+^) reducing ability of 30 μg/mL of EESB, WESB and standards is given in Table 2. Additionally, a positive relationship was found between the Cu^2+^ reducing ability and different concentration of the EESB and WESB (Figure 1B and Table 2). Cu^2+^ reducing ability of EESB and WESB was depending on concentrations (10–30 μg/mL) and demonstrated as follows: BHT (λ_450_: 1.561 ± 0.089, r^2^: 0.9978) > Ascorbic acid (λ_450_: 1.069 ± 0.007, r^2^: 0.9722) > α-Tocopherol (λ_450_: 0.785 ± 0.061, r^2^: 0.9986) > EESB (λ_450_: 0.749 ± 0.036, r^2^: 0.9970) and WESB (λ_450_: 0.441 ± 0.037, r^2^: 0.9833).

The iron chelating ability is very significant due to ionic species like ferrous ion (Fe^2+^) facilitating the generation of free ROS in the organism [71]. Metal binding activity is essential for defining and understanding the antioxidant character of a compound or crude plant extracts [18]. Fe^2+^ binding assay is a significant antioxidant method used for prevention or delaying of oxidation process catalyzed by metal ions, however excessive metal ions can cause cell damage. Among the metal ions, Fe^2+^ had the most importance and more reactive than Fe^3+^ ions. It is a significant lipid oxidizing metal due to its high activity in transition metals [72]. These reactions can also occur OH radicals, which are more reactive than the end-peroxides. Metal binding effect of EESB and WESB was evaluated using by two distinct metal chelator agents. When the IC_50_ values of the binding effect of EESB and WESB were compared with standard antioxidants, EESB was found as effective metal chelator with IC_50_: 7.2 ± 0.17 μg/mL (r^2^: 0.9296) (Figure 1E and Table 3), however, this value could not detect for WESB. Also, relatively higher IC_50_ value was found for α-Tocopherol (IC_50_: 33.0 ± 0.17 μg/mL, r^2^: 0.9109), ascorbic acid (IC_50_: 99.0 ± 0.36 μg/mL, r^2^: 0.9985) and BHT (IC_50_: 14.7 ± 0.56 μg/mL, r^2^: 0.9647).

**Table 3:** The half maximum concentration (IC_50_, μg/mL) of EESB, WESB and standards for the DPPH, and ABTS radicals scavenging activities and ferrous ion chelating ability [EESB: Evaporated ethanolic extract of aerial parts shaggy bindweed (*Convulvulus betonicifolia* Mill. Subs). WESB: Lyophilized water extract of aerial parts of shaggy bindweed (*Convulvulus betonicifolia* Mill. Subs)].

Compounds	DPPH• scavenging		ABTS^•+^scavenging		Metal chelating	
	IC_50_∗	r^2^	IC_50_∗	r^2^	IC_50_∗	r^2^
α-Tocopherol	23.1 ± 0.032	0.9825	15.4 ± 0.03	0.9866	33.0 ± 0.17	0.9109
Ascorbic acid	16.1 ± 0.03	0.9566	23.1 ± 0.01	0.9998	99.0 ± 0.36	0.9985
BHT	31.5 ± 0.01	0.9754	26.7 ± 0.08	0.9717	14.8 ± 0.56	0.9646
EESB	77.0 ± 0.01	0.9979	34.7 ± 0.02	0.9912	7.2 ± 0.17	0.9296
WESB	346.5 ± 0.02	0.9432	13.6 ± 0.03	0.9894	-∗	-∗

∗ They were not determined.

The radical scavenging is very significant in terms of damage to the organism by free radicals in living organisms. Free radicals and ROS come to the fore for accelerating lipid peroxidation. Recently, many methods have been developed for the removing of ROS and free radicals. Thus, they reduce the quality of the food and pharmaceutical products [73]. The spectrophotometric methods developed based on radical scavenging are frequently used to determine the antioxidant abilities of pure substances, beverages, food, and herbal extracts. In addition, ABTS^∙+^ and DPPH∙ scavenging methods are fast, simple, selective and repeatable procedures. So, both assays are commonly used to define the radical elimination abilities. It is easy to use the violet DPPH∙ and green-blue ABTS^∙+^ chromogens that have high sensitivity [66]. As seen in Table 3, within the scope of DPPH free radical scavenging studies, IC_50_ values for EESB and WESB had less effective DPPH∙ scavenging and were 346.5 ± 0.02 μg/mL (r^2^: 0.9432) and 77.0 ± 0.01 μg/mL (r^2^: 0.9979), respectively. Also, IC_50_ values were found as 23.1 ± 0.032 μg/mL (r^2^: 0.9825) for α-Tocopherol, 16.1 ± 0.03 μg/mL (r^2^: 0.9566) ascorbic acid, and 31.5 ± 0.01 μg/mL (r^2^: 0.9754) for BHT, which used as preservative food additive in some foods (Figure 1D and Table 3).

As with DPPH radical scavenging ability, ABTS^∙+^ scavenging ability is extensively used for determination of radical scavenging abilities of beverages, plants extract and pure molecules [40]. ABTS^∙+^ is more reactive radical than DPPH radicals. As shown Table 3, it is observed that EESB and WESB had effective ABTS radical removing effects. The IC_50_ value of ABTS^∙+^ scavenging activity for EESB and WESB was found as 34.7 ± 0.02 μg/mL (r^2^: 0.9912) and 13.6 ± 0.03 μg/mL (r^2^: 0.9894), respectively. Also, this value was recorded as 26.7 ± 0.08 μg/mL (r^2^: 0.9717) for BHT, 15.4 ± 0.03 μg/mL (r^2^: 0.9825) for α-tocopherol and 23.1 ± 0.01 μg/mL for ascorbic acid (r^2^: 0.9998). The results showed EESB and WESB have effective ABTS^∙+^ scavenging ability when compared to all standard antioxidants (Figure 1E and Table 3).

Enzyme inhibition process is one of the most common and effective strategies used in drug synthesis and design researches. Inhibition of certain metabolic enzymes can alleviate the symptoms observed in the pathology of many diseases including AD, DM and obesity [18]. In this context, AChE that had been associated in several neurodegenerative diseases including AD [74]. The AChE inhibition had positive effect on the long-term progression of AD. Also, there are many researches on the inhibition potential of pure compounds and crude extracts. One such compound is galantamine and used to treat mild AD to moderate AD [75]. Also, EESB effectively inhibited AChE with IC_50_ values of 1.946 μg/mL (r^2^: 0.9752) for AChE, which is the primary cholinesterase at mainly neuromuscular junctions and in chemical synapses in the body [76, 77]. However, it was observed that WESB had not any effects against the used metabolic enzymes.

The inhibition of AChE, α-amylase and α-glucosidase enzymes plays crucial roles in treatment of AD, hyperglycemia and DM [64]. As for DM, enzymes that hydrolyze carbohydrate polymers, namely α-glucosidase and α-amylase play very important roles for controlling of blood glucose levels [77]. Recently, DM is one of the fastest growing, serious and costly health problems in the world. The effective drugs for a complete treatment of this disease are still not available [78]. Herbal extracts and their ingredients have received great attention as safer antioxidants and potential inhibitors for important metabolic enzymes, used in clinical conditions. For example, α-glycosidase and α-amylase were essential digestive enzymes in carbohydrate metabolism and considered key targets for reducing postprandial hyperglycemia (PPG) in diabetic patients [79]. Recent researches have been conducted directly towards the discovery of α-amylase inhibitors of naturally effective ingredients and extracts with potential use as therapeutic agents for the treatment of T2DM [78]. In this context, it has been reported that biologically active compounds like acarbose, voglibose and miglitol reduce PPG by inhibiting enzymes that carry out carbohydrate digestion, thereby delaying or partially inhibiting glucose absorption [80]. However, these conventional inhibitors are associated with some undesired side effects such as bloating, abdominal pain and diarrhea [64]. EESB had marked IC_50_ values of 0.815 μg/mL (r^2^: 0.9525) toward α-glycosidase and 0.675 μg/mL against α-amylase enzymes (r^2^: 0.9706). The results show that EESB as a crude extract exhibited efficient both digestive inhibition effects than acarbose, which had IC_50_ of 10.00 mmol/L for α-amylase, 22.80 mmol/L for α-glycosidase. The results showed that EESB had more effective inhibition than that of acarbose as a starch blocker [81].

Total phenolics in EESB and WESB was determined by the Folin-Ciocalteu reagent [57]. Gallic acid, which easily obtained in large amounts by acid or alkaline hydrolysis of tannin, was used for a standard graph (r^2^: 0.9840). Plants, vegetables and fruits are important phenolic sources for human diet. Accordingly, the consumption of foods including polyphenols had a great importance for their natural antioxidant ingredients [72]. The quantity of phenolics in EESB and WESB was determined using the taken from standard gallic acid equation and found as 14.54 and 29.55 GAE/mg extract, respectively. On the other hand, 27.30 and 11.75 μg QE/mg extract flavonoids content was calculated for EESB and WESB. The most favorable structural properties that characterize the antioxidative potential of phenolic compounds are the presence of hydrogen donating substituents and the ability for delocalization of the resulting free electron for stability. The most active form of antioxidants is the one that has more than one active group like –OH in the *o*- position. In other words, the position of active groups plays an important role in the structure-activity relationship of antioxidants [44, 82]. It has been reported that the *o*- position is more active because of its ability to form intramolecular hydrogen bonds, followed by the *p*- position and followed by then *m*- position of the compounds. The hydrogen atom not involved in the intramolecular hydrogen bond is then abstracted by free radicals, resulting in the formation of a stable molecule [83]. Plants rich in phenolics therefore make a promising source of natural antioxidants. Such plants are grown commercially and used in the pharmaceutical, food and cosmetic industry, and used not only as antioxidants but also as having many biological activities [84].

Validation of the LC-HRMS method was done using analytical standards of corresponding compounds with using the target ions (Table 1) of the compounds in negative or positive ion modes and dihydrocapsaicin was used as an internal standard in the method. Validation parameters are selected as selectivity, linearity, recovery, repeatability, intermediate precision, limit of detection (LOD) and limit of quantification (LOQ) for the applied method [34]. Calibration curves were obtained from standard calibration solutions for determination of the linearity of the method for each compound. The linearity of the method was assayed by analyzing the calculation of a six-point linear plot in the range of 0.1 mg/L to 10 mg/L with six replicates. The found data of linear regression equation and the squared correlation coefficient (r^2^) were determined (Table 1) [65, 85, 86]. LOD and LOQ value of each reported compound was determined according to the following Eq. (2) (where 3 for LOQ and κ = 3 for LOD):

(2) LOD or LOQ= κSDa/b

SDa: The standard deviation of the intercept

b: Represents the slope

For recovery tests, firstly, the amount of the substances of the extract was measured by the method, later extract samples were spiked by standard solution at three concentration levels (0.5 mg/L, 1 mg/mL and 3 mg/L) in the working range and method was applied in triplicate for each level. The recovery values were calculated from following Eq. (3) and the data of them are presented in Table 1.

(3) $$\text{R}\left(\text{\%}\right)=\frac{{\text{C}}_{\text{Obs}}-{\text{C}}_{\text{Blank }}}{{\text{C}}_{\text{Spiked}}}\;\text{x}\;100$$

R(%): Recovery (%)

C_Obs_: Measured concentration of analyte by method application

C_Blank_: Concentration of analyte in the blank sample

C_Spiked_: Concentration of spiked sample  

Uncertainty parameters were determined as weighing of sampling, addition of final sample and internal stock solution, preparation of native stock solution, repeatability, recovery and calibration curve (Table 4). The detailed methodology of the uncertainty evaluation of the method are discussed in our former reports and to avoid reputation we did not discuss herein [85, 86]. The concentration of each analyte in the linear range and the concentration of the method reported were obtained from the calibration curve. The calculated concentrations were converted into mg/kg of crude sample using Eq. (4).

(4) $$\text{M}=\frac{{\text{C}}_{\text{a}}{\text{ x V}}_{\text{Final }}}{{\text{mx V}}_{\text{Initial}}}\;\text{x}\;1000$$

**Table 4:** The quantity (mg/kg extract) of phenolic compounds in EESB and WESB determined by LC-HRMS chromatograms [EESB: evaporated ethanolic extract of roots and aerial parts of Shaggy bindweed (*Convulvulus betonicifolia* Mill. Subs). WESB: Lyophilized water extract of roots and aerial parts of Shaggy bindweed (*Convulvulus betonicifolia* Mill. Subs)].

Compounds	WESB	EESB	U (%)
Ascorbic acid	139.41	24.20	3.94
(-)-Epigallocatechin	<LOD	<LOD	3.09
Chlorogenic acid	7409.23	5456.97	3.58
Verbascoside	4.62	155.63	2.93
Orientin	<LOD	10.86	3.67
Caffeic acid	149.17	105.56	3.74
Caffeic acid phenethyl ester	<LOD	0.24	3.13
(+)-*trans* Taxifolin	<LOD	5.19	3.35
Luteolin-7-rutinoside	<LOD	<LOD	3.06
Naringin	3.61	<LOD	4.20
Luteolin 7-glucoside	<LOD	10.91	4.14
Rutin	15.74	9928.10	3.07
Rosmarinic acid	71.30	5.54	3.77
Hyperoside	1864.20	389.87	3.46
Apigenin 7-glucoside	<LOD	2.28	3.59
Dihydrokaempferol	<LOD	1.89	2.86
Quercitrin	33.73	16.35	3.78
Quercetin	4.79	6.74	2.95
Salicylic acid	4.79	13.14	1.89
Naringenin	<LOD	5.15	4.20
Luteolin	0.65	3.03	3.42
Nepetin	<LOD	5.40	2.19
Apigenin	<LOD	1.97	2.87
Hispidulin	<LOD	18.30	3.41
Isosakuranetin	<LOD	<LOD	3.98
Chrysin	5.09	1.41	3.24
Acacetin	3.25	1.80	3.98
Emodin	<LOD	0.02	4.27
Fumaric acid	<LOD	4990.86	2.88

M: Amount (mg/kg)

C_a_: The concentration of compound (in μg/L)

V_Final_: Final diluted volume

M: The amount of extract as gram

V_Initial_: he initial sample volume  

According to LC-HRMS analysis, the main phenolics in 1 mg of WESB are chlorogenic acid (7409.23 mg/kg) as a polyphenolic compound that exhibits antioxidant, antibacterial and antitumor activities, hyperoside (1864.20 mg/kg) and caffeic acid (149.17 mg/kg). Besides, the high level of chlorogenic acid, hyperoside and caffeic acid in the WESB could have contributed to its antioxidant ability. On the other hand, rutin that has wide variety of medicinal applications (9928.10 mg/kg), chlorogenic acid (5456.97 mg/kg) and hyperoside (389.87 mg/kg), which as main flavonoid found in medicinal plants and had many biological activities such as antioxidant, anti-inflammatory, and cardiovascular protective effects, are the most abundant phenolic compounds in 1 mg of EESB (Table 4). It was reported that rutin was detected in the highest amount in *Silene salsuginea*. The high level of rutin, chlorogenic acid and hyperoside in the EESB could have contributed to antioxidant ability of EESB. These compounds may act synergistically with other phenolic compounds exist in EESB to produce the high antioxidant activity than that of WESB [[64], [65]]. Phenolic compounds, in other words polyphenols, are predominantly of plant origin. Plants protect themselves against many external influences. And also, the antioxidant property of polyphenols is well established [87, 88]. Phenolic compounds have different antioxidant functions such as free radical scavenger and metal chelating. In plants, the antioxidant effects of phenolics are mainly due to redox effects. For this reason, hydrogen donors, reducing agents, singlet oxygen inhibitors and metal chelates act as builders [37, 89].

## 4 Conclusions

The evaluation of the phytochemical screening and bioactivity of EESB and WESB as natural and accessible sources of polyphenolic compounds, had great importance. EESB and WESB have been found to have potent antioxidant properties in many bioanalytical tests such as Fe^3+^ and Cu^2+^ reduction abilities as well as DPPH and ABTS radical scavenging activities. Generally, the high action of the EESB in the respective assays was in proportional to its phenolic content since it was found to possess the highest total phenolic and flavonoid when compared to WESB. Indeed, many studies have also found this positive correlation between total phenolics and flavonoids of plant extracts and their radical scavenging, reducing ability and metal chelating ability. In addition, when the LC-HRMS results are evaluated, it is clearly seen that the main phenolics responsible for the antioxidant and other biological activities of both extracts are chlorogenic acid, hyperoside, and caffeic acid. Additionally, both extracts, which have potent antioxidant effects, were found to have inhibitory effects on the indicated metabolic enzymes. Also, ethanol was found efficient extraction solvent for phenolics with the effective α-glycosidase, α-amylase and AChE inhibition effects. Nowadays, the enzyme inhibition to control over active enzyme activities has become a key target in the treatment or management of many chronic diseases, especially AD, cancer and diabetes. Finally, it can be said that EESB and WESB may be a useful source of phenolic compounds that have promising potential in the treatment of some neurodegenerative and other diseases, including postural tachycardia syndrome, myasthenia gravis, diabetes and AD.

## Declarations

### Author contribution statement

Ahmet C. Gören: Conceived and designed the experiments; Performed the experiments; Wrote the paper.

Leyla Polat Kose; Meryem Topal; Lokman Durmaz: Conceived and designed the experiments; Contributed reagents, materials, analysis tools or data.

İlhami Gulcin: Conceived and designed the experiments; Wrote the paper.

Zeynebe Bingol, Hatice Kızıltaş: Performed the experiments.

Saleh H. Alwasel: Contributed reagents, materials, analysis tools or data.

### Funding statement

Saleh H. Alwasel was supported by King Saud University, Research Supporting Projects (Grant number: RSP-2021/59).

### Data availability statement

The data that has been used is confidential.

### Declaration of interests statement

The authors declare no conflict of interest.

### Additional information

No additional information is available for this paper.
